# An Anterior Cruciate Ligament Rupture Increases Levels of Urine N-terminal Cross-linked Telopeptide of Type I Collagen, Urine C-terminal Cross-linked Telopeptide of Type II Collagen, Serum Aggrecan ARGS Neoepitope, and Serum Tumor Necrosis Factor–α

**DOI:** 10.1177/03635465211042310

**Published:** 2021-09-30

**Authors:** Frans J.A. Hagemans, Staffan Larsson, Max Reijman, Richard B. Frobell, Andre Struglics, Duncan E. Meuffels

**Affiliations:** * Department of Orthopaedics and Sports Medicine, Erasmus University Medical Center, Rotterdam, the Netherlands; † Department of Orthopaedics, Center for Orthopaedic Research Alkmaar, Noordwest Ziekenhuisgroep, Alkmaar, the Netherlands; ‡ Department of Clinical Sciences, Faculty of Medicine, Lund University, Lund, Sweden; Investigation performed at the Department of Orthopaedics and Sports Medicine, Erasmus University Medical Center, Rotterdam, the Netherlands

**Keywords:** ACL injury, biomarkers, aggrecan, CTX-II, NTX-I, TNF-α

## Abstract

**Background:**

An anterior cruciate ligament (ACL) rupture results in an increased risk of developing knee osteoarthritis (OA) at an early age. Before clinical signs become apparent, the OA process has already been initiated. Therefore, it is important to look at the cascade of changes, such as the activity of cytokines and proteases, which might be associated with the later development of OA.

**Purpose:**

To compare biomarker levels in patients with a recent ACL rupture with those in controls with a healthy knee and to monitor biomarker levels over 2 years after an ACL rupture.

**Study Design:**

Descriptive laboratory study.

**Methods:**

Patients were enrolled after an ACL tear was identified. Serum and urine samples were collected at the time of enrollment in the study (3-25 weeks after the injury) and then at 14 and 27 months after the injury between January 2009 and November 2010. Reference samples were obtained from participants with healthy knees. The following biomarkers were measured with immunological assays: aggrecan ARGS neoepitope (ARGS-aggrecan), tumor necrosis factor–α (TNF-α), interferon-γ, interleukin (IL)–8, IL-10, IL-13, N-terminal cross-linked telopeptide of type I collagen (NTX-I), and C-terminal cross-linked telopeptide of type II collagen (CTX-II).

**Results:**

Samples were collected from 152 patients with an acute ACL rupture, who had a median age of 25 years (interquartile range [IQR], 21-32 years). There were 62 urine reference samples (median age, 25 years [IQR, 22-36 years]) and 26 serum reference samples (median age, 35 years [IQR, 24-39 years]). At a median of 11 weeks (IQR, 7-17 weeks) after trauma, serum levels of both ARGS-aggrecan and TNF-α were elevated 1.5-fold (*P* < .001) compared with reference samples and showed a time-dependent decrease during follow-up. Urine NTX-I and CTX-II concentrations were elevated in an early phase after trauma (1.3-fold [*P* < .001] and 3.7-fold [*P* < .001], respectively) compared with reference samples, and CTX-II levels remained elevated compared with reference samples at 2-year follow-up. Strong correlations were found between serum ARGS-aggrecan, urinary NTX-I, and urinary CTX-II (*r*_s_ = 0.57-0.68).

**Conclusion:**

In the first few months after an ACL injury, there was a measurable increase in serum levels of ARGS-aggrecan and TNF-α as well as urine levels of NTX-I and CTX-II. These markers remained high compared with those of controls with healthy knees at 2-year follow-up.

An anterior cruciate ligament (ACL) injury mostly affects the young and active population. In the short term, patients are affected by direct knee trauma in terms of pain and instability. In the long term, injured patients are at an 8-fold risk of developing knee osteoarthritis (OA).^27^ A population-based study from the United States reported an incidence of ACL injuries of 69 per 100,000 person-years, and a somewhat higher incidence of 78 per 100,000 person-years was reported in a nationwide study in Sweden.^23,26^ This shows that a large group of patients is at risk of developing knee OA at an early age. Previous studies have shown that signs of OA are present in 10% to 90% of patients at 10 to 20 years after an ACL injury.^11,18,21^ The identification of patients with early-onset OA is crucial to prevent or minimize further harm to the knee joint, and this could aid in developing treatment options for early OA. However, before clinical signs become apparent, OA has already been initiated.^1,38^ Therefore, it is important to look at the cascade of changes that occur from the moment of trauma, which might be associated with the later development of OA.

In the acute phase after knee trauma, there is an altered activity of proinflammatory cytokines and proteases, which induces an early release of C-terminal cross-linked telopeptide of type II collagen (CTX-II) and aggrecan.^17,32,33,35^ This results in structural changes of the cartilage surface and subchondral layer, which are followed by further activation of inflammatory pathways.^1,10^ At this point, OA turns into a self-perpetuating process of cartilage matrix degradation and results in irreversible damage.^30,34^ Therefore, biomarkers measuring the altered activity of proteases and inflammatory pathways may have a future role in the early identification of patients who are at risk of developing knee OA. Previous research has shown the potency of biomarkers by exploring their association with early cartilage damage and late-stage OA.^13,20,25,28^ In our previous study, we reported increased levels of synovial fluid proinflammatory cytokines and the aggrecan ARGS neoepitope (ARGS-aggrecan) within the first weeks after an injury and prolonged alterations of tumor necrosis factor–α (TNF-α) up to 5 years after trauma.^32^ Also, concentrations of urinary CTX-II were highly increased early after an injury, while urine N-terminal cross-linked telopeptide of type I collagen (NTX-I) levels were not. These findings indicated early damage of the knee joint and an elevated state of inflammation from the moment of trauma. To date, this is, to our knowledge, the only study measuring a comprehensive set of biomarkers in a large longitudinal cohort of patients with an ACL injury.^32^ To verify these findings, more longitudinal studies with large cohorts are necessary. Therefore, in the present study, we tested a similar set of biomarkers in urine and serum of patients with an ACL injury in a larger sample size than previously published.^32^ The primary aim was to compare biomarker levels of patients with a recent ACL rupture to those of controls with a healthy knee. Secondarily, we aimed to investigate the biomarker levels over a 2-year time period after an ACL rupture.

## Methods

### Patients and Biosamples of the ACL Injury Group

**Figure 1.**
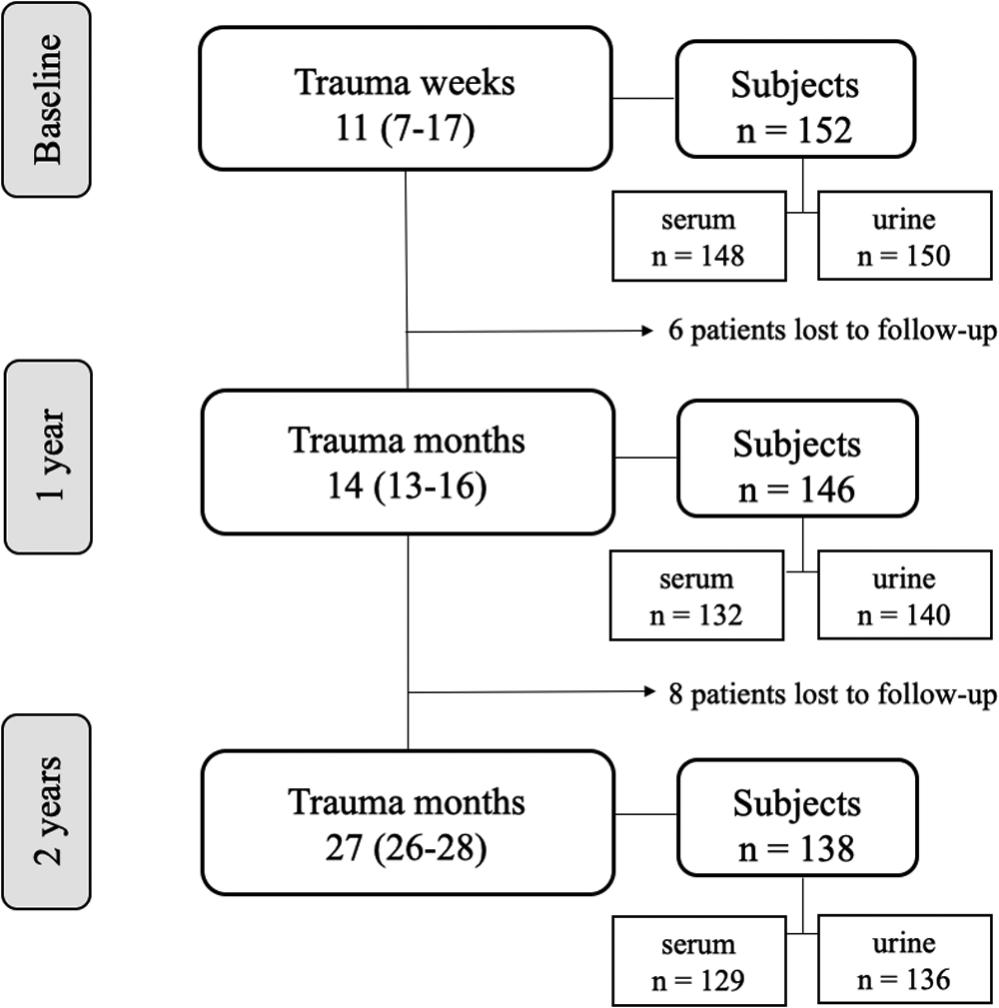
Flow diagram of time points, study participants, and biosamples. Weeks and months after trauma are presented as the median with the interquartile range in parentheses.

From the KNALL (Knee Osteoarthritis Anterior Cruciate Ligament Lesion) cohort, this prospective observational study included 152 patients with an ACL rupture (Figure 1).^39^ Sampling for this study was conducted between January 2009 and November 2010 at 3 hospitals in the Netherlands: Erasmus University Medical Center in Rotterdam, Medical Center Haaglanden in The Hague, and Reinier de Graaf Gasthuis in Delft. The study was approved by the ethical review board, and all patients gave their informed consent. The patients were aged between 18 and 45 years and were diagnosed with an ACL rupture via a physical examination and magnetic resonance imaging. Patients were excluded if they had a previous ACL injury or meniscal and/or cartilage damage and/or OA changes on radiographs (Kellgren and Lawrence grade >0); inclusion and exclusion criteria have been previously reported.^40^ The patients in the study were either treated operatively or nonoperatively, according to the practical guidelines of the Dutch Orthopaedic Association,^22^ and followed for 2 years after the injury. Urine and serum samples were collected at the time of enrollment in the study (3-25 weeks after the injury) and at 14 months (interquartile range [IQR], 13-16 months) and 27 months (IQR, 26-28 months) after the injury (Table 1 and Figure 1).

**Table 1:** Characteristics of Participants^*a*^

ACL Injury Group	n	Age, Median (IQR), y	Female Sex, n (%)	Time From Trauma to Surgery, Median (IQR), wk	
Total	152	25 (21-32)	52 (34)	—	
Nonoperative treatment	54	30 (24-30)	20 (37)	—	
Surgery	98	23 (20-28)	31 (32)	24 (14-36)	
Reference Group	n	Age, Median (IQR), y	*P* Value^*b*^	Female Sex, %	*P* Value^*c*^
Serum					
ARGS-aggrecan	26	35 (24-39)	**.006**	39	.626
Cytokines	16^*d*^	26 (21-36)	.740	44	.414
Urine					
CTX-II/NTX-I	62	25 (22-36)	.174	27	.382

a Statistical significance between groups is shown in boldface. ACL, anterior cruciate ligament; ARGS-aggrecan, aggrecan ARGS neoepitope; CTX-II, C-terminal cross-linked telopeptide of type II collagen; IQR, interquartile range; NTX-I, N-terminal cross-linked telopeptide of type I collagen; —, not applicable.

b The Mann-Whitney *U* test was used for a comparison of age between the groups.

c The chi-square test was used for a comparison of sex between the groups.

d A total of 16 of the 26 reference samples used previously^32^ were reanalyzed in this study and used as a reference for cytokine comparisons.

### Participants and Biosamples of the Reference Group

Overall, 3 types of samples from participants with healthy knees were used as references in the molecular biomarker analyses. Characteristics of the reference participants and their biosamples are presented in Table 1.

For ARGS-aggrecan analysis, serum samples from 26 participants with healthy knees (based on normal findings on clinical examination, arthroscopic surgery, and/or radiography) were from a cross-sectional cohort included in our previous study.^32^ These participants were older compared with patients in the KNALL cohort (Table 1).

For cytokine analyses, serum samples were obtained from 16 of 26 participants with healthy knees (because of sample depletion). These samples were from the same cross-sectional cohort as was included for ARGS-aggrecan analysis (Table 1).

For NTX-I and CTX-II analyses, urine samples were used from 62 participants with healthy knees without arthritis or a history of joint injuries, surgery, or symptoms. NTX-I and CTX-II analyses of the urine samples were performed in our previous study.^32^

All biosamples were stored at −80°C until analysis, and all biomarker analyses were conducted at the Biomedical Centre at Lund University in Sweden.

### ARGS-aggrecan

A sandwich immunoassay using the Meso Scale Discovery platform (Meso Scale Diagnostics) was performed for the detection of ARGS-aggrecan in serum.^14^ Samples were primarily assayed at final dilutions of 1:2.6, and if needed, samples were assayed in dilutions of 1:1.3, 1:5.2, or 1:9.1 in duplicate. Of the 409 samples, 1 sample was missed because of sample depletion; thus, 408 samples were analyzed in the ARGS-aggrecan assay. One sample had a concentration below the lower limit of quantification (LLOQ; ie, 0.025 pmol/mL)^14^ and was therefore imputed given the concentration of half the LLOQ (0.0125 pmol/mL). The technical performance of the ARGS-aggrecan assay in this study is presented in Appendix Table A1 (see the Appendix, available in the online version of this article). Samples of the ACL injury group and reference group were measured at 2 different occasions using 2 different lot numbers of the capture antibody. Because there is a lot dependency in the assay, we conducted (1) a direct lot-to-lot comparison of results on 4 different serum samples on the 2 lots in the same experiment and (2) a reanalysis of 2 samples from the ACL injury group on the lot used for the reference samples. Results on the lot used for the ACL injury group were, on average, 1.503 times higher than results on the lot used for the reference group. Therefore, reference data for ARGS-aggrecan were multiplied with 1.503 for comparison with the ACL injury group.

### Cytokines

To determine proinflammatory cytokines in serum, we used the V-PLEX Proinflammatory Panel 1 Human Kit (No. K15049D-1; Meso Scale Diagnostics) with plates analyzed in a Sector Imager 6000 (Meso Scale Diagnostics). This panel included interferon (IFN)–γ, interleukin (IL)–1β, IL-2, IL-4, IL-6, IL-8, IL-10, IL-12p70, IL-13, and TNF-α. Samples from the ACL injury group and the 16 reference samples were measured using the proinflammatory panel at the same occasion using the same assay lot number. In accordance with the manufacturer’s protocol, serum samples were thawed and centrifuged at 2000*g* for 3 minutes before the assessments. To optimize the amount of measurable cytokine markers, we analyzed the serum samples undiluted in duplicate. Despite measuring samples undiluted, we found a large proportion (ie, 67%-100% of samples) of IL-12p70, IL-1β, IL-2, IL-4, and IL-6 had concentrations below their LLOQs and/or their mean coefficients of variation (CVs) were high (ie, ≥20%), and therefore, these cytokine markers were excluded from further analysis (Appendix Table A2, available online). The technical performance of cytokines used in further analyses is presented in Appendix Table A1.

### NTX-I and CTX-II

The urine samples were thawed and centrifuged at 3000 ×*g* for 10 minutes, collecting supernatants before being assayed.

Urine concentrations of NTX-I were measured (according to the manufacturer’s protocol) using the Osteomark NTx urine immunoassay (No. 9006; Alere). The urine samples were analyzed undiluted in duplicate, although samples with values above the upper limit of detection were rerun using a 1:5 dilution. Data from the NTX-I assay are expressed as nanomoles of NTX-I (bone collagen equivalents [BCE]) per millimole of creatinine. Of the 426 samples analyzed using the NTX-I assay in this study, 5 samples were below the LLOQ and were therefore imputed with half the LLOQ (ie, 32.5 nM BCE).

Urine concentrations of CTX-II were measured (according to the manufacturer’s protocol) using the Urine CartiLaps immunoassay (No. AC-10F1; Immunodiagnostic Systems). The urine samples were analyzed at a dilution of 1:2 in duplicate, although samples with values under the lower limit of detection (LLOD) were rerun undiluted, and samples above the upper limit of detection were rerun in dilutions between 1:5 and 1:100. Data from the CTX-II assay are expressed as nanograms of CTX-II per millimole of creatinine. Of the 426 samples analyzed using the CTX-II assay in this study, 33 samples had values below the LLOQ but above the LLOD; because of satisfactory CVs between duplicates (ie, <20%), we decided to use these data for further analysis. A total of 9 samples had values below the LLOD, and for these, the values were imputed with half the LLOD (ie, 0.10 µg/L creatinine).

We used 2 different lot numbers for both the NTX-I and CTX-II assays, although according to the manufacturers, there are no batch-to-batch differences in these assays. The technical performance of the NTX-I and CTX-II assays is presented in Appendix Table A1.

Urine concentrations of creatinine (CREP2; Roche Diagnostics) were measured at the Department of Clinical Chemistry, Office of Medical Services Region Skâne, Sweden.

### Statistical Analysis

The LLOQ and upper limit of quantification for each cytokine and collagen biomarker were calculated from the standard curves of all plates, identifying the lowest and highest standards for which recovery was between 80% and 120%, with a CV between duplicates of <20% (Appendix Tables A1 and A2, available online). For the NTX-I and cytokine assays, samples with concentrations below the LLOQ were imputed using half the concentration of the corresponding LLOQ value. For the CTX-II assay, values below the LLOQ but above the LLOD were used, and values below the LLOD were imputed using half the concentration of the LLOD (Appendix Table A1). None of the biomarker data were normally distributed (assessed via the Shapiro-Wilk test and via inspections of histograms and boxplots); therefore, nonparametric statistical tests were used. For baseline characteristics, group differences in age were assessed via the Mann-Whitney *U* test, and the chi-square test was used to assess group differences in sex. To determine whether the biomarker concentrations of the patients with ACL injuries were elevated compared with the reference samples, the Mann-Whitney *U* test was used. Generalized estimating equation (GEE) models were used to study time-dependent differences in biomarker concentration, with biomarker concentrations as the dependent variable and the time points during follow-up as the covariate. Additionally, GEE models were adjusted by adding age at baseline as a covariate. Results are expressed as the regression coefficient (B) and 95% confidence interval of the parameter estimates. Also, a subgroup analysis was conducted for the treatment of ACL injuries (surgery or nonoperative therapy) by means of GEE models, and when significant differences were present, the Mann-Whitney *U* test was performed to compare between time points. The Spearman rank correlation was used to determine correlations between biomarkers at baseline. To assess whether our results were influenced by the imputation of values below the LLOQ, we conducted a sensitivity analysis in which (1) data were imputed with zero, (2) data were imputed with the LLOQ, or (3) all imputed values were removed. *P* values <.05 were considered as statistically significant. Statistical analysis was performed using SPSS Statistics (Version 24; IBM Corp).

## Results

### Serum ARGS-aggrecan Levels Were Elevated at Baseline and at 1- and 2-Year Follow-up

**Figure 2.**
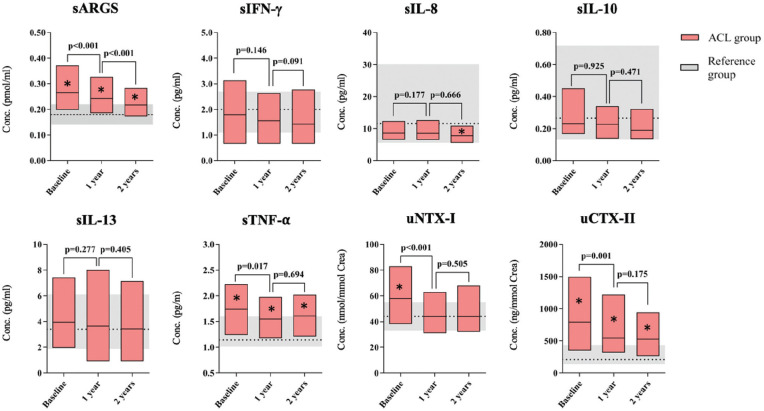
Graphic view of biomarkers. Data are presented as the median with interquartile range. Statistical differences between the anterior cruciate ligament (ACL) injury group and the reference group are indicated with an asterisk (*). *P* values are presented for significant decreases in marker concentration within the ACL injury group between time points according to generalized estimating equation analysis. Conc., concentration; ARGS-aggrecan, aggrecan ARGS neoepitope; CTX-II, C-terminal cross-linked telopeptide of type II collagen; IFN, interferon; IL, interleukin; NTX-I, N-terminal cross-linked telopeptide of type I collagen; s, serum; TNF-α, tumor necrosis factor–α; u, urine.

At baseline, the median serum ARGS-aggrecan concentration of the ACL injury group was elevated 1.5-fold (*P* < .001) compared with that of the reference group, and even though concentrations had decreased over the 2-year period, their levels were still 1.2 times higher than the reference levels (*P* = .010) (Figure 2 and Table 2). For the ACL injury group, over the 2-year period, ARGS-aggrecan levels decreased by 0.05 pmol/mL (corresponding to 19% from baseline) (Figure 2 and Table 2). At baseline, serum ARGS-aggrecan of the ACL injury group showed a strong positive correlation with urine CTX-II (*r*_s_ = 0.682) and NTX-I (*r*_s_ = 0.570) (Table 3).

**Table 2:** Data Overview of Serum and Urine Biomarkers^*a*^

Serum ARGS-aggrecan	Concentration, pmol/mL	Norm	*P* Value	N (n With Data > LLOQ)
Reference group	0.18 (0.14-0.22)	1.00	—	26 (26)
ACL injury group				
Baseline	0.27 (0.20-0.36)	1.50	**<.001**	147 (146)^*b*^
1 y	0.24 (0.19-0.33)	1.33	**<.001**	132 (132)
2 y	0.22 (0.17-0.28)	1.22	**.010**	129 (129)
Serum IFN-γ	Concentration, pg/mL	Norm	*P* Value	N (n With Data > LLOQ)
Reference group	2.01 (0.66-2.71)	1.00	—	16 (11)
ACL injury group				
Baseline	1.79 (0.66-3.13)	0.89	.910	148 (93)
1 y	1.56 (0.66-2.64)	0.78	.634	132 (73)
2 y	1.43 (0.66-2.77)	0.71	.504	129 (69)
Serum IL-8	Concentration, pg/mL	Norm	*P* Value	N (n With Data > LLOQ)
Reference group	11.64 (5.57-30.10)	1.00	—	16 (16)
ACL injury group				
Baseline	8.60 (6.59-12.33)	0.74	.175	148 (148)
1 y	8.52 (6.52-12.65)	0.73	.135	132 (132)
2 y	7.76 (5.59-10.87)	0.73	**.042**	129 (129)
Serum IL-10	Concentration, pg/mL	Norm	*P* Value	N (n With Data > LLOQ)
Reference group	0.26 (0.13-0.72)	1.00	—	16 (16)
ACL injury group				
Baseline	0.23 (0.17-0.45)	0.88	.731	148 (140)
1 y	0.23 (0.14-0.34)	0.88	.333	132 (113)
2 y	0.19 (0.14-0.32)	0.73	.244	129 (118)
Serum IL-13	Concentration, pg/mL	Norm	*P* Value	N (n With Data > LLOQ)
Reference group	3.41 (1.23-6.11)	1.00	—	16 (12)
ACL injury group				
Baseline	3.94 (1.95-7.42)	1.16	.510	148 (112)
1 y	3.65 (0.93-8.01)	1.07	.685	132 (95)
2 y	3.44 (0.93-7.14)	1.01	.794	129 (94)
Serum TNF-α	Concentration, pg/mL	Norm	*P* Value	N (n With Data > LLOQ)
Reference group	1.14 (1.01-1.60)	1.00	—	16 (16)
ACL injury group				
Baseline	1.74 (1.24-2.23)	1.53	**<.001**	148 (147)
1 y	1.55 (1.18-1.98)	1.36	**.010**	132 (131)
2 y	1.61 (1.21-2.02)	1.41	**.005**	129 (127)
Urine NTX-I	Concentration, nmol/mmol Creatinine	Norm	*P* Value	N (n With Data > LLOQ)
Reference group	45 (33-55)	1.00	—	62 (52)
ACL injury group				
Baseline	58 (38-83)	1.29	**<.001**	150 (149)
1 y	44 (31-63)	0.98	.589	140 (139)
2 y	44 (32-68)	0.98	.519	136 (133)
Urine CTX-II	Concentration, ng/mmol Creatinine	Norm	*P* Value	N (n With Data > LLOQ)
Reference group	212 (136-437)	1.00	—	62 (52)
ACL injury group				
Baseline	791 (350-1498)	3.73	**<.001**	150 (149)
1 y	544 (319-1220)	2.57	**<.001**	140 (138)
2 y	526 (262-942)	2.48	**<.001**	136 (130)

a Data are presented as the median (interquartile range) unless otherwise specified. The Mann-Whitney *U* test was used for comparisons between the ACL injury and reference groups. *P* values were unadjusted for multiple testing. Statistical significance between groups is shown in boldface. ACL, anterior cruciate ligament; ARGS-aggrecan, aggrecan ARGS neoepitope; CTX-II, C-terminal cross-linked telopeptide of type II collagen; IFN, interferon; IL, interleukin; LLOQ, lower limit of quantification; NTX-I, N-terminal cross-linked telopeptide of type I collagen; TNF-α, tumor necrosis factor–α.

b Data from 1 patient were missing because of sample depletion.

**Table 3:** Correlation Analyses Between Biomarkers at Baseline of the ACL Injury Group^*a*^

	Serum ARGS-aggrecan (1% < LLOQ)	Serum IFN-γ (37% < LLOQ)	Serum IL-8 (0% < LLOQ)	Serum IL-10 (5% < LLOQ)	Serum IL-13 (24% < LLOQ)	Serum TNF-α (1% < LLOQ)	Urine NTX-I (1% < LLOQ)	Urine CTX-II (1% < LLOQ)	Strength of Correlation
Serum ARGS-aggrecan	—	−0.055 (.510)	−0.024 (.769)	0.166 **(.044)**	0.035 (.678)	0.163 **(.049)**	0.570 **(<.001)**	0.682 **(<.001)**	NS
Serum IFN-γ		—	0.203 **(.014)**	0.446 **(<.001)**	0.131 (.112)	0.362 **(<.001)**	−0.070 (.402)	−0.045 (.584)	>0.1
Serum IL-8			—	0.151 (.067)	0.081 (.330)	0.287 **(<.001)**	0.002 (.978)	−0.069 (.407)	>0.2
Serum IL-10				—	0.275 **(.001)**	0.554 **(<.001)**	−0.054 (.513)	0.086 (.299)	>0.3
Serum IL-13					—	0.241 **(.003)**	−0.033 (.687)	0.035 (.673)	>0.4
Serum TNF-α						—	0.001 (.995)	0.062 (.458)	>0.5
Urine NTX-I							—	0.653 **(<.001)**	>0.6

a Data are presented as the Spearman rank correlation with *P* values in parentheses. The number of observations in the correlation analyses was between 146 and 150. Statistically significant correlations are shown in boldface, with the strength of the correlations indicated. ARGS-aggrecan, aggrecan ARGS neoepitope; CTX-II, C-terminal cross-linked telopeptide of type II collagen; IFN, interferon; IL, interleukin; LLOQ, lower limit of quantification; NS, not significant; NTX-I, N-terminal cross-linked telopeptide of type I collagen; TNF-α, tumor necrosis factor–α.

### Serum TNF-α Levels Were Elevated at Baseline and at 1- and 2-Year Follow-up

At baseline, serum TNF-α in the ACL injury group was the only cytokine elevated compared with in the reference group (1.5-fold; *P* < .001); TNF-α levels of the ACL injury group decreased but were still 1.4-fold higher (*P* = .005) compared with those in the reference group at 2 years after the injury (Table 2 and Figure 2). For the ACL injury group, over the 2-year period, TNF-α levels decreased by 0.13 pg/mL (corresponding to 7% from baseline) (Figure 2 and Table 2).

The serum IL-8 concentration at 2-year follow-up was 1.4-fold lower (*P* = .042) compared with reference levels (Table 2 and Figure 2). The sensitivity analysis showed similar results for these comparisons (Appendix Table A3, available online).

For the ACL injury group at baseline, several of the serum cytokines showed a positive correlation with each other, whereas TNF-α correlated to all the other cytokines (Table 3).

### Urine NTX-I Was Elevated at Baseline, and Urine CTX-II Was Elevated at Baseline and at 1- and 2-Year Follow-up

At baseline, urine NTX-I and CTX-II concentrations in the ACL injury group were elevated (1.3-fold [*P* < .001] and 3.7-fold [*P* < .001], respectively) compared with those in the reference group (Table 2 and Figure 2). While the concentration of urinary CTX-II of the ACL injury group was still elevated 2.5-fold (*P* < .001) at 2-year follow-up, urine NTX-I levels were normalized to the levels of the reference group within a year from the injury. For the ACL injury group, over the 2-year period, the median NTX-I and CTX-II levels decreased by 14 nmol/mmol of creatinine (corresponding to 24% of baseline) and 265 ng/mmol of creatinine (corresponding to 31% of baseline), respectively (Figure 2 and Table 2). There was a strong positive correlation between the 2 collagen markers at baseline (Table 3).

### For the ACL Injury Group, Increasing Age at Baseline Was Associated With Decreasing Serum ARGS-aggrecan and Urine CTX-II and NTX-I Concentrations

For the ACL injury group, age and time after trauma both had a negative effect on ARGS-aggrecan concentrations; however, the effect of time was stronger than the effect of age (Table 4). For NTX-I and CTX-II, similar effects were observed; age and time both had a negative effect on marker concentrations, while the effect of time was stronger than the effect of age (Table 4). No association was found between serum cytokine levels and age and time after trauma for the ACL injury group during follow-up (data not shown).

**Table 4:** Interactions of Time and Age With Biomarker Concentrations of the ACL Injury Group^*a*^

	B (95% CI)	*P* Value
Serum ARGS-aggrecan		
Time	−0.132 (−0.182 to −0.081)	**<.001**
Age at baseline	−0.015 (−0.020 to −0.010)	**<.001**
Urine NTX-I		
Time	−36 (−53 to −18)	**<.001**
Age at baseline	−4 (−6 to −3)	**<.001**
Urine CTX-II		
Time	−2150 (−3370 to −930)	**.001**
Age at baseline	−234 (−342 to −126)	**<.001**

a Only markers with a significant interaction with age are shown. ACL, anterior cruciate ligament; ARGS-aggrecan, aggrecan ARGS neoepitope; CTX-II, C-terminal cross-linked telopeptide of type II collagen; NTX-I, N-terminal cross-linked telopeptide of type I collagen.

### Patients Who Were Treated With ACL Reconstruction Had Higher Levels of Serum ARGS-aggrecan and Urine NTX-I and CTX-II at Baseline and at 2-Year Follow-up

Patients in the surgery group were treated with ACL reconstruction at a median of 24 weeks (IQR, 14-36 weeks) after trauma. Concentrations of serum ARGS-aggrecan were higher in the surgery group at baseline (*P* = .002) and at 2 years of follow-up (*P* = .016) (Table 5). Also, concentrations of ARGS-aggrecan showed a steeper decline (*P* = .015) within the first year of follow-up compared with those in the nonoperative group. Concentrations of urinary NTX-I and CTX-II in the surgery group were both elevated compared with those in the nonoperative group at all 3 time points (*P* < .010) (Table 5). Concentrations of CTX-II in the surgery group showed a steeper decline (*P* = .038) in the first year of follow-up compared with those in the nonoperative group. There were no differences in the concentrations of cytokines.

**Table 5:** Subgroup Analysis of the ACL Injury Group^*a*^

	Baseline		1 y		2 y	
	Surgery Subgroup	Nonoperative Subgroup	Surgery Subgroup	Nonoperative Subgroup	Surgery Subgroup	Nonoperative Subgroup
Serum ARGS-aggrecan, pmol/mL	0.29 (0.22-0.40)^*b*^ [n = 94]	0.23 (0.18-0.31) [n = 53]	0.25 (0.19-0.34) [n = 88]	0.22 (0.17-0.29) [n = 44]	0.22 (0.18-0.29)^*b*^ [n = 83]	0.21 (0.14-0.24) [n = 46]
Serum IFN-γ, pg/mL	1.83 (0.66-3.13) [n = 95]	1.76 (0.66-3.18) [n = 53]	1.45 (0.66-2.61) [n = 88]	1.87 (0.66-2.96) [n = 44]	1.36 (0.66-2.89) [n = 83]	1.57 (0.66-2.66) [n = 46]
Serum IL-8, pg/mL	8.28 (6.50-11.26) [n = 95]	9.54 (6.80-13.38) [n = 53]	8.06 (6.47-11.80) [n = 88]	9.37 (6.59-14.25) [n = 44]	7.37 (5.56-10.14) [n = 83]	7.83 (5.58-12.59) [n = 46]
Serum IL-10, pg/mL	0.23 (1.17-0.45) [n = 95]	0.22 (0.17-0.47) [n = 53]	0.24 (0.15-0.35) [n = 88]	0.22 (0.12-0.31) [n = 44]	0.20 (0.13-0.36) [n = 83]	0.18 (0.14-0.28) [n = 46]
Serum IL-13, pg/mL	3.91 (0.93-9.90) [n = 95]	4.38 (2.06-6.11) [n = 53]	3.65 (0.92-8.71) [n = 88]	3.78 (0.93-6.09) [n = 44]	3.49 (0.93-9.18) [n = 83]	3.44 (1.64-6.51) [n = 46]
Serum TNF-α, pg/mL	1.68 (1.18-2.14) [n = 95]	1.82 (1.46-2.39) [n = 53]	1.49 (1.18-1.96) [n = 88]	1.68 (1.08-2.03) [n = 44]	1.57 (1.12-2.12) [n = 83]	1.66 (1.38-2.00) [n = 46]
Urine NTX-I, nmol/mmol creatinine	62 (41-91)^*b*^ [n = 96]	52 (37-67) [n = 54]	50 (33-69)^*b*^ [n = 92]	36 (28-50) [n = 48]	45 (33-74)^*b*^ [n = 87]	40 (27-55) [n = 49]
Urine CTX-II, ng/mmol creatinine	906 (446-1821)^*b*^ [n = 96]	417 (249-1083) [n = 54]	740 (410-1456)^*b*^ [n = 92]	365 (133-649) [n = 48]	655 (382-1002)^*b*^ [n = 87]	395 (151-584) [n = 49]

a Data are presented as the median (interquartile range). ACL, anterior cruciate ligament; ARGS-aggrecan, aggrecan ARGS neoepitope; CTX-II, C-terminal cross-linked telopeptide of type II collagen; IFN, interferon; IL, interleukin; NTX-I, N-terminal cross-linked telopeptide of type I collagen; TNF-α, tumor necrosis factor–α.

b Statistically significant differences at the corresponding follow-up point.

## Discussion

In this study, patients with a recent ACL rupture had increased serum concentrations of ARGS-aggrecan and TNF-α compared with the reference group with healthy knees. At 2 years after trauma, the concentrations of ARGS-aggrecan and TNF-α had decreased but were still elevated compared with those in the reference group. Also, urine concentrations of CTX-II and NTX-I were elevated after trauma compared with those in the reference group, with a subsequent decrease over time, although CTX-II but not NTX-I remained higher at 2-year follow-up compared with levels observed in the reference group.

We found a 1.5-fold elevation in serum ARGS-aggrecan levels within the baseline period of 3 to 25 weeks after trauma, which corroborates with findings in our previous study in which we found a 1.4-fold increase within the baseline period of 0 to 6 weeks after trauma.^32^ Both our studies showed a similar decrease in concentration during 2-year follow-up, which is also in accordance with earlier observations.^15,31,37^ Thus, ARGS-aggrecan is involved in the early phase after trauma.

Our elevated concentrations of serum TNF-α compared with levels from reference samples were inconsistent with our previous study^32^ in which we found equal levels of serum TNF-α at all time points compared with those of the reference group. An explanation might be that the TNF-α assay used for this study was more sensitive (LLOD = 0.04 pg/mL) than the one used previously (LLOD = 0.28 pg/mL),^32^ although, in that study, we did not have any data below the LLOQ for serum TNF-α. Nonetheless, our increased levels of serum TNF-α showed similarities with the elevation and concomitant decrease of TNF-α concentrations in synovial fluid found previously.^15^ Other studies have investigated TNF-α in synovial fluid and established its role in local knee joint inflammation and its interaction with proteoglycans and other cytokines.^20,24^ However, few studies have reported about TNF-α in serum, and to our knowledge, no study has found elevated levels of serum TNF-α after knee trauma.^3,29^ The study of Stannus et al^29^ revealed an association of serum TNF-α with tibial cartilage loss and medial joint space narrowing in an older patient population. It seems possible that serum TNF-α may function as an inflammatory mediator in an early stage after trauma.

A weak correlation was found between serum TNF-α and ARGS-aggrecan. According to Dahlberg et al,^7^ aggrecan fragments were found to be elevated in injured as well as uninjured knees after a knee injury. This finding supports the idea that there is an enhanced systemic reaction after knee trauma in which TNF-α might play a role.

Furthermore, compared with those in the reference group, concentrations of serum IL-8 were decreased at 2-year follow-up, which is in concordance with our previous study.^15^ IL-8 is known as a regulator in the inflammatory process of OA, and it is induced by TNF-α.^9,12,19^ Further work is needed to explore the role of serum TNF-α and IL-8 in knee injuries and in OA.

The elevated levels of urinary CTX-II at baseline are consistent with our earlier study.^32^ Both studies revealed an increase of urinary CTX-II, followed by a decline years thereafter. This was also seen in the study of Chmielewski et al,^6^ who followed patients with an ACL rupture up to 16 weeks after trauma. The elevated levels of CTX-II fragments indicated the remodeling or breakdown of cartilage type II collagen shortly after trauma, similarly as seen for ARGS-aggrecan in this study. It was somewhat surprising that our urine NTX-I levels were increased after trauma, as we did not find an elevation in our previous study.^32^ However, both studies did show a decrease with increasing time after an injury. This may be associated with increased bone marrow lesion volumes observed shortly after an ACL injury or decreasing bone mineral density during the first year of follow-up.^8,40^ Several reports have shown the potential of NTX-I as a marker for radiographic OA in a later stage of disease^2,42^; this early increase of NTX-I indicates altered bone turnover also shortly after trauma. This could be attributed to increased remodeling and bone loss in an early stage, before late-stage remodeling and subchondral densification take place.^4^ This hypothesis is supported by lower bone mineral density levels in injured knees compared with contralateral knees of the KNALL cohort found in a previous study.^40^ Further work is needed to clarify the role of NTX-I in an early phase after trauma.

Consistent with the literature, this study showed a positive correlation between urinary NTX-I and CTX-II.^32,36^ The study of van Spil et al^41^ hypothesized that CTX-II measured in urine may merely reflect bone turnover rather than cartilage degradation based on stronger associations with bone markers than cartilage markers, while others have stated that CTX-II originates primarily from cartilage.^5^ Nonetheless, we found a correlation between urinary CTX-II and both serum ARGS-aggrecan and urinary NTX-I, which might suggest that CTX-II reflects both cartilage degradation and bone turnover.

In our subgroup analysis, concentrations of serum ARGS-aggrecan and urinary NTX-I and CTX-II were higher for the surgery group compared with the nonoperative group. We did not observe a trauma-induced effect in the cytokines, contrary to the study of Larsson et al,^16^ who found elevated levels of IL-6, IL-8, and TNF-α in synovial fluid at up to 8 months of follow-up in patients treated with ACL reconstruction. This may be explained by the fact that our cytokines were measured within a wider time frame and in serum instead of synovial fluid. Lower concentrations of ARGS-aggrecan, CTX-II, and NTX-I in the nonoperative group may partially be explained by an older age compared with that of the surgery group (30 vs 23 years, respectively) (Table 1). It is also possible that higher marker concentrations resulted from more severe bone bruises and damage to the (sub)chondral layer at initial trauma or from repetitive microtrauma caused by instability and that those patients were more likely to undergo ACL reconstruction. This hypothesis is supported by the study of Frobell et al,^8^ who found a prolonged resolution time of bone marrow lesions and greater changes in cartilage morphometry within the first year after trauma for patients who underwent ACL reconstruction. Furthermore, it can be argued whether higher levels of NTX-I at baseline are a result of disuse in the preoperative phase. The role of ACL treatment on biomarker levels is not entirely understood at present.

The strengths of our study include the large patient population, multiple follow-up time points, a wide spectrum of biomarkers, and the ability to compare our data with that of a reference group. The time between trauma and the collection of samples was, for some patients, several weeks, with a median of 11 weeks after trauma. This was because of an inclusion period of up to half a year after trauma and is in accordance with the ACL guidelines in the Netherlands.^22^ Therefore, we have limited data within the first week(s) after trauma, and it can be argued whether we have missed elevated levels of cytokines in the acute phase after trauma because previous work has shown that cytokines are induced locally within days after an injury and show a rapid decrease during follow-up.^32,35^ Despite a high sensitivity of the V-PLEX Proinflammatory Panel 1 Human Kit, a large proportion of the data (>67%) was below the LLOQ for some inflammatory markers (IL-1β, IL-2, IL-4, IL-6, and IL-12p70) and therefore excluded from further analysis (Appendix Table A2, available online). For some markers (ie, IL-10 and IFN-γ), substantially more samples had concentrations above the LLOQ in the V-PLEX Proinflammatory Panel 1 Human Kit compared with the Human Proinflammatory 7-Plex kit (Meso Scale Diagnostics) (91% vs 56%, respectively, >LLOQ for IL-10 and 57% vs 19%, respectively, >LLOQ for IFN-γ) used in our previous study. For IL-13 and IFN-γ, 26% and 42%, respectively, of concentrations were below the LLOQ. The comparison of serum ARGS-aggrecan of patients with an ACL injury with that of participants with healthy knees should be interpreted carefully because there was an age difference between groups and the reference data were produced on a different lot of the capture antibody. However, the conversion of the old data based on direct lot-to-lot comparisons resulted in similar differences between the ACL injury and reference groups, as noted in our previous study,^32^ which indicated that the compensation made for lot change was credible. Finally, the sample size of the reference group was smaller than that of the ACL injury group. Despite an older age on ARGS-aggrecan analysis for the reference group, patient characteristics were comparable. The sample size of cytokines was limited to 16 samples; therefore, the median and IQR of the controls may approach the mean concentration of a healthy population less precisely, especially for markers with merely imputed data. For this reason, our comparisons of cytokines with the reference group have to be interpreted with caution.

In summary, in the first months after an ACL rupture, there was a measurable increase in serum ARGS-aggrecan and TNF-α as well as urinary NTX-I and CTX-II. These markers remained relatively high compared with those of healthy controls during follow-up, which might be the result of a prolonged state of inflammation with an increased activity of cartilage proteases. Further work is required to establish the clinical value of the investigated markers, which reflect early changes within the joint.

## Supplemental Material

sj-pdf-1-ajs-10.1177_03635465211042310 – Supplemental material for An Anterior Cruciate Ligament Rupture Increases Levels of Urine N-terminal Cross-linked Telopeptide of Type I Collagen, Urine C-terminal Cross-linked Telopeptide of Type II Collagen, Serum Aggrecan ARGS Neoepitope, and Serum Tumor Necrosis Factor–α Click here for additional data file. Supplemental material, sj-pdf-1-ajs-10.1177_03635465211042310 for An Anterior Cruciate Ligament Rupture Increases Levels of Urine N-terminal Cross-linked Telopeptide of Type I Collagen, Urine C-terminal Cross-linked Telopeptide of Type II Collagen, Serum Aggrecan ARGS Neoepitope, and Serum Tumor Necrosis Factor–α by Frans J.A. Hagemans, Staffan Larsson, Max Reijman, Richard B. Frobell, Andre Struglics and Duncan E. Meuffels in The American Journal of Sports Medicine
