# Supplementary material for: An Anterior Cruciate Ligament Rupture Increases Levels of Urine N-terminal Cross-linked Telopeptide of Type I Collagen, Urine C-terminal Cross-linked Telopeptide of Type II Collagen, Serum Aggrecan ARGS Neoepitope, and Serum Tumor Necrosis Factor–α
Source: Am J Sports Med. 2021 Sep 30;49(13):3534–43. doi: 10.1177/03635465211042310 (PMC8573615; doi:10.1177/03635465211042310)
Supplement: sj-pdf-1-ajs-10.1177_03635465211042310 – Supplemental material for An Anterior Cruciate Ligament Rupture Increases Levels of Urine N-terminal Cross-linked Telopeptide of Type I Collagen, Urine C-terminal Cross-linked Telopeptide of Type II Collagen, Serum Aggrecan ARGS Neoepitope, and Serum Tumor Ne [file sj-pdf-1-ajs-10.1177_03635465211042310.pdf]

Anterior cruciate ligament rupture increases levels of urine N- and C-terminal crosslinked telopeptides of type I and II collagens, serum aggrecan ARGS neoepitope and tumor necrosis factor-α.

Appendix

Table A1. Technical performance of assays and samples used in this study.

	sARGS	sIFN-γ	sIL-8	sIL-10	sIL-13	sTNF-α	uNTX-I	uCTX-II
**Assay range:**								
LLOD	0.010 pmol/ml	0.42 pg/ml	0.09 pg/ml	0.06 pg/ml	1.10 pg/ml	0.12 pg/ml	30 nmol/L	0.20 μg/L
LLOQ	0.025 pmol/ml	1.328 pg/ml	0.563 pg/ml	0.097 pg/ml	1.867 pg/ml	0.355 pg/ml	65 nmol/L	0.64 μg/L
ULOQ	0.200 pmol/ml	1360 pg/ml	576 pg/ml	359 pg/ml	476 pg/ml	364 pg/ml	3000 nmol/L	8.67 μg/L
**[1]Inter assay CV, % (n):**								
Serum control	8.38 (12)	11.44 (12)	10.3 (12)	8.2 (12)	15.7 (12)	16.2 (12)	-	-
High control	-	-	-	-	-	-	8.4 (13)	6.0 (15)
Low control	-	-	-	-	-	-	8.5 (13)	7.9 (14)
**Intra assay CV %, mean (min, max):**								
Serum control	2.6 (0.6, 3.9)	4.5 (0.0, 12.5)	2.6 (0.5, 5.2)	2.2 (0.2, 9.1)	1.5 (0.3, 3.8)	4.0 (0.2, 9.8)	-	-
High control	-	-	-	-	-	-	8.3 (0.53, 33.2)	6.3 (0.21, 0.0)
Low control	-	-	-	-	-	-	5.8 (0.58, 10.0)	7.0 (0.8, 15.4)
**Sample data > LLOQ or LLOD, n (%)**	407*(100%)	235 (57%)	409 (100%)	371 (91%)	301 (74%)	405 (99%)	421 (99%)	417 (98%)
**Sample CV:**								
Mean, % (n)	2.1 (407)	6.3 (235)	2.1 (409)	5.3 (371)	3.5 (301)	4.0 (405)	7.5 (421)	7.6 (417)
Min, max in %	0.0, 15.3	0.0, 31.4	0.0, 16.7	0.0, 20.6	0.0, 29.7	0.0, 26.3	0.0, 32.9	0.0, 22.6

(1) For the NTX-I and CTX-II biomarkers the inter and intra assay coefficient of variation (CV) were calculated for the manufacturers high and low sample controls, for ARGS-aggrecan and cytokine markers this was done for an in-house serum control sample. The lower limit of quantification (LLOQ) and upper limit of quantification (ULOQ) were calculated for each assay based on standard and sample performance of all tested plates. Sample data above LLOQ for all biomarkers except for CTX-II which shows data above LLOD. *Data from one patient was missed due to sample depletion. CTX-II = C-terminal crosslinking telopeptide of type II collagen; IFN = interferon; IL = interleukin; LLOD = lower limit of detection; NTX-I = N-terminal crosslinking telopeptide of type I collagen; TNF = tumour necrosis factor; s = serum; u = urine.

Table A2. Technical performance of assays and samples that were not further analyzed in this study.

	sIL-1β	sIL-2	sIL-4	sIL-6	sIL-12p70
**Assay range, pmol/ml:**					
LLOD	0.054	0.127	0.017	0.085	0.130
LLOQ	0.137	1.396	0.060	0.681	0.118
ULOQ	140.25	357.50	61.00	174.25	120.50
**Inter assay CV, % (n):**					
In-house serum control	24.0 (12)	22.3 (12)	16.7 (12)	18.8 (12)	13.3 (12)
**Intra assay CV %, mean (min, max):**					
In-house serum control	7.1	4.3	5.1	2.9	3.7
**Sample data > LLOQ, n (%)**	30 (7%)	0 (0%)	89 (22%)	43 (11%)	133 (33%)
**Sample CV:**					
Mean, % (n)	6.4 (30)	n/a	4.9 (89)	2.5 (43)	9.5 (133)

CV = coefficient of variation (CV); IL = interleukin; LLOD = lower limit of detection; LLOQ = lower limit of quantification; ULOQ = upper limit of quantification; s = serum; n/a = not applicable.

Table A3. Sensitivity analysis for the value of imputated data.

**sARGS**		*Imputation of data with 0.025*	*Imputation of data with 0.000*	
	**Group analysis**	**P-values**	**P-values**	**N (n data > LLOQ)**
				26 (26)
	Reference versus			
	Baseline	**<0.001**	**<0.001**	147 (146)[*]
	1 year	**<0.001**	**<0.001**	132 (132)
	2 years	**0.010**	**0.010**	129 (129)
**sIFN-γ**		*Imputation of data with 1.328*	*Imputation of data with 0.000*	
	**Group analysis**	**P-values**	**P-values**	**N (n data > LLOQ)**
			-	16 (11)
	Reference versus			
	Baseline	0.910	0.910	148 (93)
	1 year	0.634	0.634	132 (73)
	2 years	0.504	0.504	129 (69)
**sIL-8**		*Imputation of data with 0.563 pg/ml*	*Imputation of data with 0.000*	
	**Group analysis**	**P-values**	**P-values**	**N (n data > LLOQ)**
			-	16 (16)
	Reference versus			
	Baseline	0.175	0.175	148 (148)
	1 year	0.135	0.135	132 (132)
	2 years	**0.042**	**0.042**	129 (129)
**sIL-10**		*Imputation of data with 0.097 pg/ml*	*Imputation of data with 0.000*	
	**Group analysis**	**P-values**	**P value**	**N (n data > LLOQ)**
			-	16 (16)
	Reference versus			
	Baseline	0.731	0.731	148 (140)
	1 year	0.333	0.333	132 (113)

	2 years	0.244	0.244	129 (118)
**sIL-13**		*Imputation of data with 1.867*	*Imputation of data with 0.000*	
	**Group analysis**	**P-values**	**P-values**	**N (n data > LLOQ)**
			-	16 (12)
	Reference versus			
	Baseline	0.510	0.510	148 (112)
	1 year	0.685	0.685	132 (95)
	2 years	0.794	0.794	129 (94)
**sTNFα**		*Imputation of data with 0.355*	*Imputation of data with 0.000*	
	**Group analysis**	**P-values**	**P-values**	**N (n data > LLOQ)**
			-	16 (16)
	Reference versus			
	Baseline	**<0.001**	**<0.001**	148 (147)
	1 year	**0.010**	**0.010**	132 (131)
	2 years	**0.005**	**0.005**	129 (127)
**uNTX-I**		*Imputation of data with 65*	*Imputation of data with 0.000*	
	**Group analysis**	**P-values**	**P-values**	**N (n data > LLOQ)**
			-	62 (52)
	Reference versus			
	Baseline	**<0.001**	**<0.001**	150 (149)
	1 year	0.609	0.609	140 (139)
	2 years	0.537	0.537	136 (133)
**uCTX-II**		*Imputation of data with 0.64*	*Imputation of data with 0.000*	
	**Group analysis**	**P-values**	**P-values**	**N (n data > LLOQ)**
				62 (52)
	Reference versus			
	Baseline	**<0.001**	**<0.001**	150 (149)
	1 year	**<0.001**	**<0.001**	140 (138)
	2 years	**<0.001**	**<0.001**	136 (130)

Median (interquartile range) concentrations of biomarkers are presented. P-values are presented from Mann-Whitney tests for comparisons between the ACL and the reference groups. *Data from one patient was missed due to sample depletion. Statistical significance between subject groups are shown in boldface. ARGS = aggrecan ARGS neoepitope; CTX-II = C-terminal crosslinked telopeptide of type II collagen; IFN = interferon; NTX-I = N-terminal crosslinked telopeptide of type I collagen; IL = interleukin; s = serum; TNF = tumor necrosis factor; u = urine.
